# Using physiology to understand climate-driven changes in disease and their implications for conservation

**DOI:** 10.1093/conphys/cot022

**Published:** 2013-08-26

**Authors:** Jason R. Rohr, Thomas R. Raffel, Andrew R. Blaustein, Pieter T. J. Johnson, Sara H. Paull, Suzanne Young

**Affiliations:** 1 Integrative Biology, University of South Florida, Tampa, FL 33620, USA; 2 Department of Biological Science, Oakland University, Rochester, MI 48309-4401, USA; 3 Department of Zoology, Oregon State University, Corvallis, OR 97331-2914, USA; 4 Department of Ecology and Evolutionary Biology, University of Colorado, Boulder, CO 80309-0334, USA

**Keywords:** Acclimatization, amphibian, chytridiomycosis, climate change, host–parasite interaction, metabolic theory of ecology

## Abstract

Controversy persists regarding the contributions of climate change to biodiversity losses, through its effects on the spread and emergence of infectious diseases. One of the reasons for this controversy is that there are few mechanistic studies that explore the links among climate change, infectious disease, and declines of host populations. Given that host–parasite interactions are generally mediated by physiological responses, we submit that physiological models could facilitate the prediction of how host–parasite interactions will respond to climate change, and might offer theoretical and terminological cohesion that has been lacking in the climate change–disease literature. We stress that much of the work on how climate influences host–parasite interactions has emphasized changes in climatic means, despite a hallmark of climate change being changes in climatic variability and extremes. Owing to this gap, we highlight how temporal variability in weather, coupled with non-linearities in responses to mean climate, can be used to predict the effects of climate on host–parasite interactions. We also discuss the climate variability hypothesis for disease-related declines, which posits that increased unpredictable temperature variability might provide a temporary advantage to pathogens because they are smaller and have faster metabolisms than their hosts, allowing more rapid acclimatization following a temperature shift. In support of these hypotheses, we provide case studies on the role of climatic variability in host population declines associated with the emergence of the infectious diseases chytridiomycosis, withering syndrome, and malaria. Finally, we present a mathematical model that provides the scaffolding to integrate metabolic theory, physiological mechanisms, and large-scale spatiotemporal processes to predict how simultaneous changes in climatic means, variances, and extremes will affect host–parasite interactions. However, several outstanding questions remain to be answered before investigators can accurately predict how changes in climatic means and variances will affect infectious diseases and the conservation status of host populations.

## Abstract (precis)

Given that host-parasite interactions are generally mediated by physiological responses, we submit that physiological models could facilitate predicting how host-parasite interactions respond to climate change, and might offer theoretical and terminological cohesion that has been lacking in the climate change-disease literature.

## Introduction

Over the past 50 years, scientists have documented significant anthropogenic climate change and extraordinary biodiversity losses (Walther *et al.*, 2002; Stuart *et al.*, 2004; Thomas *et al.*, 2004; Parmesan, 2006). Anthropogenic greenhouse gas emissions are affecting several components of climate, including temperature and precipitation means, variances, and extremes (Easterling *et al.*, 2000; Meehl *et al.*, 2000, 2009; Raisanen, 2002; Kunkel *et al.*, 2003). Both paleontological and contemporary data suggest that such changes in temperature and precipitation can contribute to declines in biodiversity (Walther *et al.*, 2002; Stuart *et al.*, 2004; Thomas *et al.*, 2004; Parmesan, 2006). Indeed, there is evidence that recent climate changes have already caused population declines or extirpations of lizards, corals, butterflies, and polar bears (Stirling *et al.*, 1999; Bruno *et al.*, 2007; Sinervo *et al.*, 2010; Breed *et al.*, 2013). Moreover, projected climate-induced faunal changes suggest profound changes in populations of numerous species (Lawler *et al.*, 2009).

Alongside changes in climate, scientists have also documented unprecedented rates of emerging infectious diseases over the last 50 years (Daszak *et al.*, 2000; Stuart *et al.*, 2004; Thomas *et al.*, 2004; Jones *et al.*, 2008; Lafferty, 2009; Rohr *et al.*, 2011a). Although it was traditionally assumed that parasites did not cause extinctions of their host populations because parasites themselves require a threshold host population size to persist, we know now that there are several conditions in which parasites can cause host population extinctions, such as when the parasite can persist in alternative hosts or environmental reservoirs (de Castro and Bolker, 2005). Even if these conditions do not occur, parasites can drive host populations to low levels and, coupled with demographic stochasticity, can contribute to host extinctions (de Castro and Bolker, 2005). For example, emerging infections have been linked to population declines of chestnut, elm, pine, and oak trees (Anagnostakis, 1987; Mamiya, 1988; Brasier, 2001; Rizzo and Garbelotto, 2003; Anderson *et al.*, 2004; Rohr *et al.*, 2011a). Emerging fungal infections are driving losses of amphibians, snakes, bees, crayfish, and bats (McCallum *et al.*, 2009; Rohr *et al.*, 2011a; Fisher *et al.*, 2012). Malaria is believed to have caused declines in the diversity of Hawaiian birds (Vanriper *et al.*, 1986; Garamszegi, 2011), and an infectious cancer has caused the near extinction of the Tasmanian devil (McCallum *et al.*, 2009; Rohr *et al.*, 2011a; Fisher *et al.*, 2012). Consequently, increases in infectious diseases often threaten biodiversity.

Several researchers have suggested that climate change is indirectly contributing to biodiversity losses by increasing the spread and emergence of infectious diseases (Epstein, 2000; Harvell *et al.*, 2002; Lafferty, 2009; Rohr *et al.*, 2011a). Nonetheless, the extent to which climate change causes increases in some diseases but declines in others is an area of active debate, and more studies are required to identify the diseases that will be likely to pose the greatest risk in the event of future climate change (Lafferty, 2009; Rohr *et al.*, 2011a). Mechanistic studies of how climate change influences disease will help clarify this debate for several reasons. First, mechanisms provide concrete evidence of a cause-and-effect relationship between a change in climate and an increase in infectious disease. Such evidence is urgently needed to enable identification of the diseases, regions, and species most likely to face increased disease threats with climate change (Rohr *et al.*, 2011a). Second, our predictions for how host–parasite interactions will respond to shifts in climate should improve if we understand the underlying mechanisms linking these factors. Finally, understanding the mechanistic links between climate and disease should allow us to develop more effective measures targeted at mitigating the threats posed to species by pathogens.

**Figure 1:**
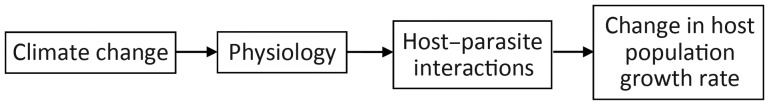
Flowchart highlighting the importance of physiology in understanding the influence of climate on host–parasite interactions and changes in host population densities.

Virtually all host–parasite interactions are mediated by physiological responses, and climate can have profound effects on these responses, especially for ectothermic species, which comprise 99.9% of all species (Daufresne *et al.*, 2009). Therefore, understanding physiology is essential for attaining a mechanistic understanding of how climate change affects infectious disease (Fig. 1). Physiology has a rich history of quantifying both the response of biological and chemical systems to mean climatic conditions (*Q*_10_ coefficients) and the response of organisms to climatic variability through quantification of acclimatization effects (Hutchison, 1961; Brattstrom, 1963; Spotila, 1972; Nadel *et al.*, 1974; Atkin and Tjoelker, 2003; Hoffmann *et al.*, 2003). Hence, physiological models should facilitate the prediction of how host–parasite interactions respond to simultaneous changes in climatic means, variances, and extremes, and might offer theoretical and terminological cohesion that has been lacking in the climate change–disease literature (Martin *et al.*, 2010; Blaustein *et al.*, 2012).

The goal of this paper is to explore how physiology and disease ecology can be better integrated to understand the outcome of climate–disease interactions, especially those that involve species of conservation concern. We first offer background on the physiology and thermal biology of host–parasite interactions. We stress that much of the work on how climate influences host–parasite interactions has emphasized changes in climate means, despite a hallmark of climate change being alterations in climate variability and extremes (Easterling *et al.*, 2000; Meehl *et al.*, 2000, 2009; Raisanen, 2002; Kunkel *et al.*, 2003). Owing to this gap in the literature, we highlight the role of climatic variability in driving host–parasite interactions. Specifically, we discuss how non-linearities in response to mean climate, coupled with temporal variability in weather and organismal acclimatization responses, can be used to predict the effects of climate on host–parasite interactions. We propose that the metabolic theory of ecology (MTE) offers an instrument to integrate physiological mechanisms and large-scale spatiotemporal processes to enable successful prediction of how changes to climatic means, variances, and extremes will affect host–parasite interactions. We then provide a quantitative example for how metabolic theory can be used to derive models of host–parasite interactions in a changing temperature environment. Finally, we end with conclusions and outstanding questions.

## Thermal biology and disease

In this section, we explore the application of approaches from thermal biology to host–parasite interactions. We begin by defining performance curves and show how they can be used to predict host–parasite interactions, highlighting differences in the temperature-dependent defense strategies of ecto- and endothermic hosts. We then discuss the importance of phenotypic plasticity of hosts and parasites to temporal variability in temperature, emphasizing likely differences in the acclimatization rates of hosts and parasites.

### Thermal performance curves of parasites and ecto- and endothermic hosts

**Figure 2:**
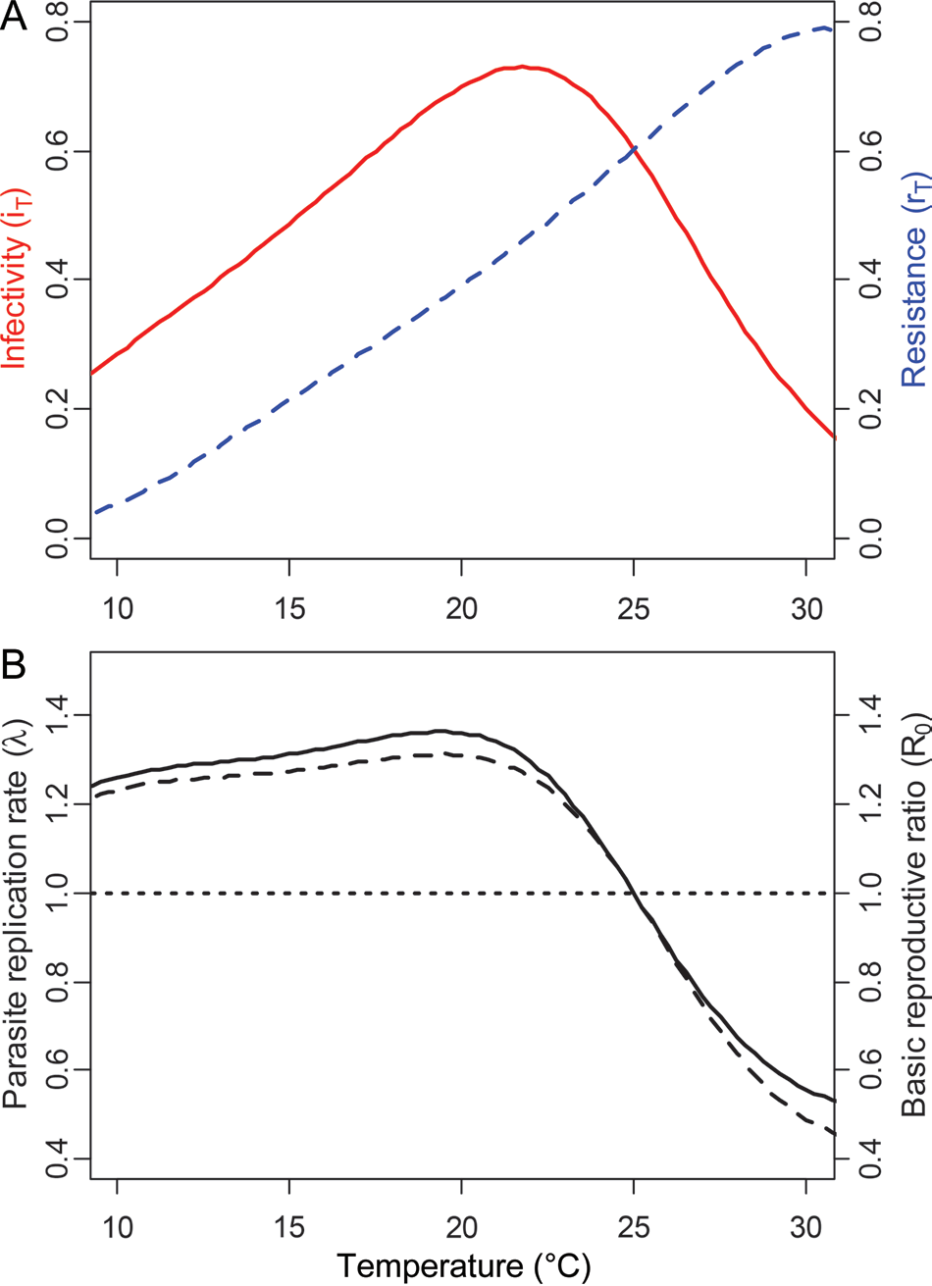
Model predictions for temperature dependence of *Batrachochytrium dendrobatidis* infection on Cuban treefrogs (*Osteopilus septentrionalis*). (A) Performance curves of *B. dendrobatidis* infectivity (continuous red line) and host resistance (dashed blue line) as functions of temperature. (B) Temperature dependence of the within-host parasite replication rate, λ (continuous line), and the basic reproductive ratio for disease transmission, *R*_0_ (dashed line), in a population of 500 susceptible hosts. The within-host parasite replication rate (λ) is a function of parasite infectivity (intrinsic population growth rate of parasite within host) and host resistance (decrease in parasite growth rate as a result of host immune responses), and *R*_0_ is a function of λ. See main text for model equations and parameter values.

Performance curves depict the ability of an organism to perform a physiological function (e.g. muscle strength) across a range of temperatures (Angilletta *et al.*, 2002; Angilletta, 2009). In host–parasite interactions, the physiological parameters of most interest are the rate of growth or replication of the parasite within or on the host (referred to here as ‘parasite infectivity’) and the capacity of the host to reduce parasite growth (referred to here as ‘host resistance’; Raffel *et al.*, 2013). We can conceptualize these two processes as separate thermal performance curves, the combination of which describes the ability of the parasite to infect the host at a particular temperature (Fig. 2A and B). Both parasite infectivity and host resistance may be temperature dependent, so both must be accounted for when predicting the effects of temperature on infection (Raffel *et al.*, 2013).

There might also be fundamental differences in the thermal biology of parasitism in ectothermic vs. endothermic hosts, the latter of which has received more attention by ecological immunologists (Martin *et al.*, 2008). The thermal environment of a parasite should be essentially constant within an endothermic host, which expends energy heating or cooling itself to maintain a constant internal temperature. Thus, effects of external temperature on the parasites of an endotherm might be indirect and driven primarily by energetic trade-offs between host thermoregulation and investment in immune responses (Nelson and Demas, 1996). This leads to a general prediction of decreased host resistance to infection when external temperatures are far from the host's optimal temperature, because an endotherm would need to invest more in maintaining its internal temperature, thereby leaving less energy available to invest in anti-parasite defenses. However, many endothermic hosts have parasites transmitted by ectothermic vectors, thus making many of the concepts discussed below regarding ectothermic hosts relevant to the transmission of vector-borne parasites to endothermic hosts.

Parasites of ectothermic hosts might often experience different thermal environments from parasites of endothermic hosts, because the internal temperature of ectothermic hosts varies with external temperature (Angilletta, 2009). Thus, both the host and the parasite must adapt to temperature at the tissue level (Raffel *et al.*, 2013). Furthermore, lower temperatures will not necessarily drive energetic trade-offs between thermoregulation and the host immune response; indeed, many ectotherms expend less energy at lower temperatures because their metabolic rates are reduced (Gillooly *et al.*, 2001). Depending on their specific thermal optima relative to hosts, pathogen and parasite growth rates might also decrease at lower temperatures (Ratkowsky *et al.*, 1982), such that a slower immune response might be sufficient to control infection (Raffel *et al.*, 2006, 2013; Murdock *et al.*, 2012). Thus, the effects of temperature on the parasites of ectothermic hosts should depend on the thermal performance curves of both parasite growth (Fig. 2) and host resistance and might be driven less by energetic trade-offs with host thermoregulation than they are for endothermic hosts. Importantly, much of the work in thermal biology has been conducted on organismal responses to mean temperature, despite climate change also affecting temporal variability in temperature (Easterling *et al.*, 2000; Benedetti-Cecchi, 2003).

### Host and parasite phenotypic plasticity in response to temporal variability in temperature

Thermal biology also provides insights into potential effects of sudden shifts in temperature on host–parasite interactions. Biologists have long known that organisms can respond to a temperature shift by adjusting their physiological systems to operate more effectively at the new temperature (Angilletta, 2009). Such plastic responses to temperature shifts are referred to as physiological acclimatization, which can be thought of as the organism shifting its performance curve to optimize performance at the new temperature (Angilletta, 2009). Acclimatization responses are usually assumed to be adaptive, but they are energetically costly and take time (Deere and Chown, 2006). Therefore, acclimatization responses are effective only if an organism can expect to be at the new temperature for an extended period of time, or if it can anticipate when the next temperature shift will occur. Thus, the frequency and predictability of temperature variation are likely to determine the effectiveness of acclimatization responses in both free-living and parasitic organisms (Raffel *et al.*, 2013).

Thermal acclimatization responses are potentially important for understanding the effects of temperature variability on infectious disease, because parasites and hosts are likely to have differential responses to temperature variability. For example, metabolic theory predicts that smaller organisms will have faster metabolisms and correspondingly shorter times to complete physiological processes, including thermal acclimatization (Pörtner, 2002); therefore, parasites, which are almost universally smaller than their hosts, would be expected to acclimatize to new temperatures more rapidly than their hosts (Raffel *et al.*, 2013). Indeed, it can take weeks for ectothermic vertebrates to acclimatize their immune systems to a new temperature (Bly and Clem, 1991), whereas one of the few parasites investigated so far acclimatized to comparable temperature shifts in a matter of hours (Terblanche and Chown, 2006). Therefore, parasites might become more infectious in conditions of frequent and unpredictable shifts in temperature, because of lags in host acclimatization following temperature shifts (Raffel *et al.*, 2006). This hypothesis has potentially important implications for responses of host–parasite systems to climate change, which is altering temperature variability over various time scales (Easterling *et al.*, 2000; Meehl *et al.*, 2000, 2009; Raisanen, 2002; Kunkel *et al.*, 2003).

Hosts can also use plastic responses, such as behaviours, to track temperatures ideally suited for resisting infections, a process called behavioural thermoregulation (Angilletta, 2009; Kearney *et al.*, 2009). Ectothermic hosts often use behaviour to increase body temperature in response to infectious diseases (‘behavioural fever’; Lefcort and Blaustein, 1995; Thomas and Blanford, 2003; Elliot *et al.*, 2005) and might also use behavioural thermoregulation to modulate temporal shifts in temperature (e.g. to move to a relatively warm place if the temperature drops). Spatial variation in temperature can also assist endothermic hosts in responding to pathogens by allowing them to reduce the energetic costs of physiological thermoregulation. For example, bats that gather in warmer parts of caves during winter are better able to survive infection by the fungus that causes white-nose syndrome, because they expend less energy heating their bodies in response to the pathogen (Boyles and Willis, 2010). Behavioural thermoregulation also poses an empirical challenge, however, because it suggests that coarse-scale temperature projections might sometimes fail to capture the local temperatures experienced by particular hosts or pathogens (Boyles and Willis, 2010). The extent to which such behavioural effects might diminish the predictive power of models based on coarse-scale temperatures is unclear, however, because large-scale temperature shifts might cause losses of suitable habitat or overwhelm the capacity of organisms to thermoregulate, even in heterogeneous environments. Thus, further study is needed to determine the extent to which behavioural thermoregulation can help hosts mitigate effects of climate shifts on infection.

## Effects of climate change on host–parasite interactions

Climate change involves changes to both the mean and the variability of climatic conditions. Here, we discuss evidence in support of the hypothesis that shifts to both temperature means and variances affect host–parasite interactions and that these altered interactions are facilitating some host population declines. We argue for development of models that integrate the effects of changes to both temperature means and variances on host–parasite interactions to distribute limited conservation funds effectively.

### Mean temperature effects

The outcomes of host–parasite interactions in different constant-temperature conditions in the laboratory are generally straightforward to predict if one has the appropriate physiological temperature-dependent performance curves of the host and the parasite. For instance, if a shift in mean temperature from 10 to 12°C causes a greater increase in relevant performance metrics of the parasite relative to the host, then this increase in mean temperature should disproportionately benefit the parasite in this interaction. Indeed, several theoretical, observational, and experimental studies have demonstrated tight linkages between host and parasite performance metrics and changes in mean climatic conditions (Altizer *et al.*, 2006). For instance, climate has been shown to have particularly strong effects on pathogens with free-living environmental stages (e.g. *Vibrio* spp.) and ectothermic vectors (e.g. *Plasmodium* spp.; Pascual *et al.*, 2008; Paaijmans *et al.*, 2009; Murdock *et al.*, 2012). These types of pathogens often have pronounced inter-annual and seasonal fluctuations that correlate well with changes in temperature or precipitation (Altizer *et al.*, 2006; Pascual *et al.*, 2008), and which can be predicted using climate-based models (Paaijmans *et al.*, 2010).

**Figure 3:**
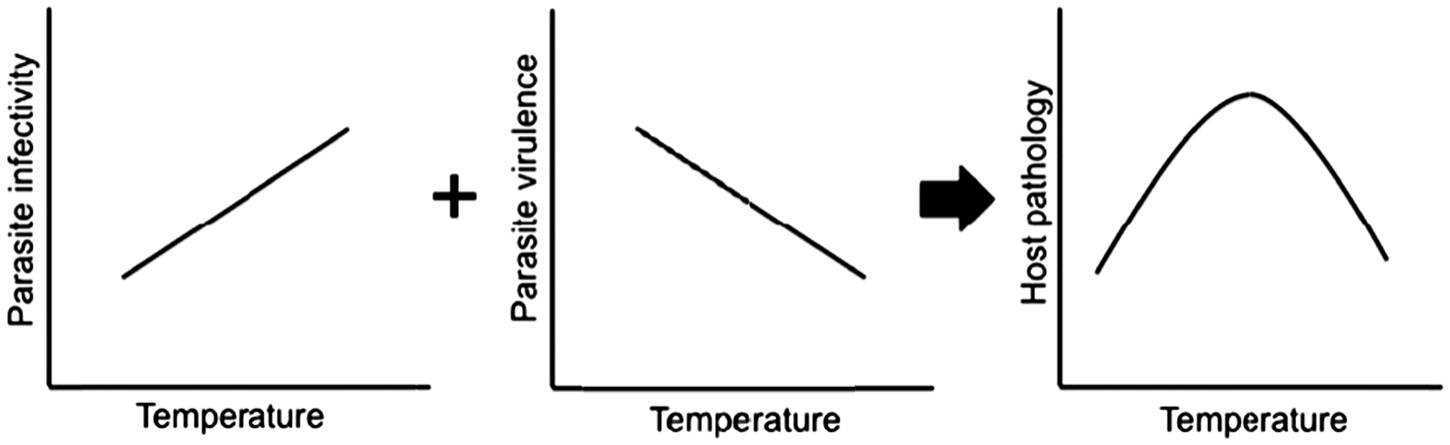
Hypothetical diagram showing that increases in parasite infectivity that are approximately linear over a small range of temperatures combined with linear declines in parasite virulence or host susceptibility (potentially caused, for example, by faster host growth out of vulnerable stages of development) can yield non-linear changes to host pathology.

Growing evidence, however, suggests that disease risk may relate non-linearly to temperature (Rohr *et al.*, 2011a; Paull *et al.*, 2012), which can complicate efforts to predict how host–parasite interactions will respond to shifts in mean temperature. Several recent studies have demonstrated non-linear relationships between the basic reproductive rate of a parasite (*R*_0_) and temperature, stemming from underlying non-linearities in parasite and/or vector performance (Molnár *et al.*, 2013; Mordecai *et al.*, 2013). Non-linearities can arise in several ways. First, host, vector, and/or parasite performances across temperature might be inherently non-linear, as was shown for *Plasmodium* spp. and the amphibian chytrid fungus, *Batrachochytrium dendrobatidis* (Paaijmans *et al.*, 2009, 2010; Mordecai *et al.*, 2013; Raffel *et al.*, 2013). Alternatively, non-linearities in disease risk may arise as a result of integration across generally linear portions of host and parasite temperature performance curves that have opposite slopes (Fig. 3). This latter mechanism reflects the fact that disease is the product of interactions between two species—host and pathogen—each of which may have their own responses to temperature change. Both mechanisms of generating non-linearities have been shown to be important for pathogens causing species extinctions (Box 1). Moreover, assumptions of linearity alter the predictions for how climate change would affect disease and thus extinction risk, illustrating the conservation importance of integrating the non-linear physiological responses of both host and parasite to temperature (Box 1).

### The importance of temperature variability

**Figure 4:**
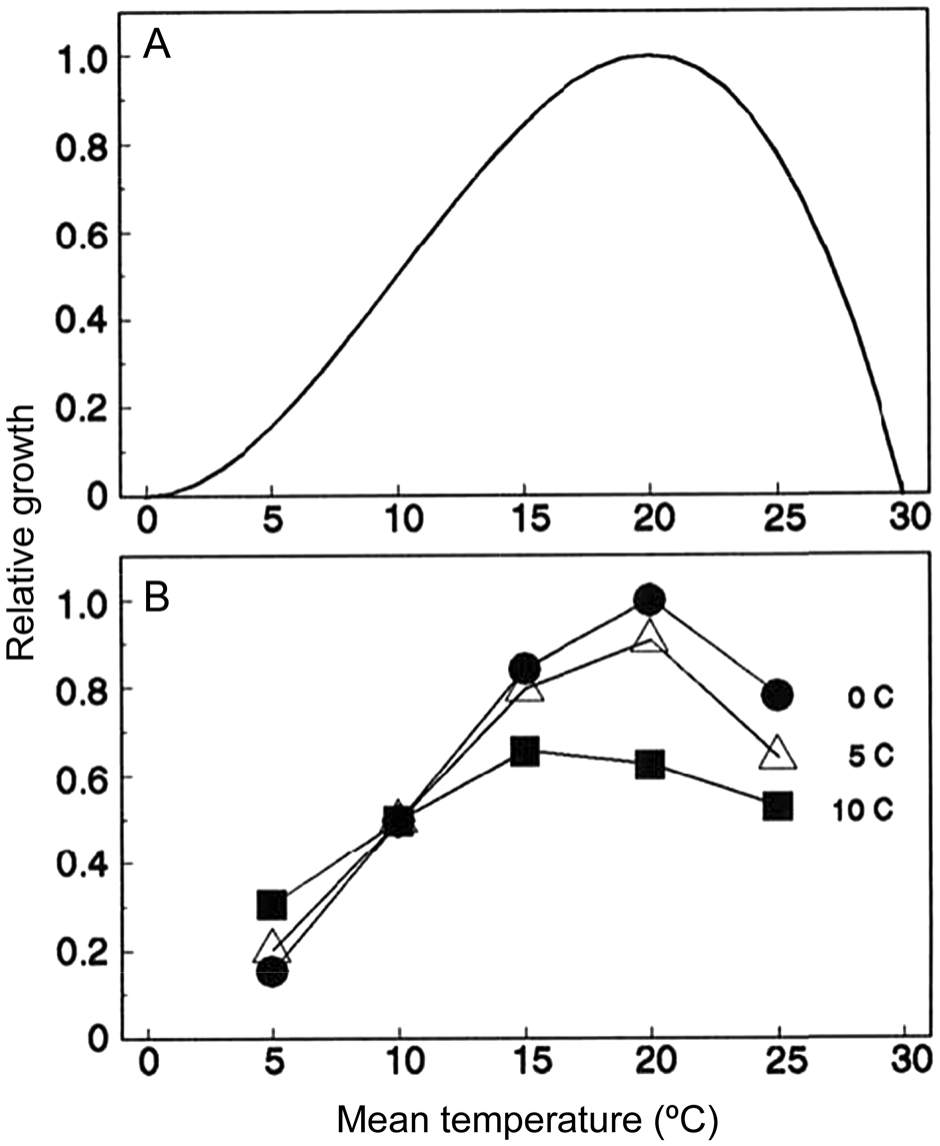
Simulated growth curves for a hypothetical pest species at constant temperatures (A) or at variable temperatures (B) with sinusoidal fluctuations of various amplitudes (0°C, circles; 5°C, diamonds; and 10°C, squares). Reprinted from Scherm H and van Bruggen AHC (1994) Global warming and non-linear growth: how important are changes in average temperature? Phytopathology 84: 1380–1384, with permission from The American Phytopathological Society.

The incorporation of non-linearities in disease risk into climate-driven disease models becomes even more important in the context of temperature variability (Scherm and van Bruggen, 1994; Raffel *et al.*, 2013). This is because when a response to temperature is purely linear, then the only information required to predict the average response accurately is the average temperature. However, when responses to temperature are non-linear, as is almost always the case if a wide enough thermal range is considered, it is necessary to know how much time was spent at each temperature to predict the response accurately, because estimates based on mean temperature will yield erroneous results. Scherm and van Bruggen (1994) illustrated this phenomenon using simulated performance curves for growth of a hypothetical pest species with a thermal optimum between 25 and −30°C (Fig. 4A). Using performance curves to calculate growth rates at a variety of simulated temperatures and levels of fluctuation, the authors show that the greater the amplitude of the temperature fluctuation, the more the growth predicted by the mean temperature deviates from that predicted by the performance curve at a given temperature (Fig. 4B). For instance, a temperature fluctuation of 15°C around a mean temperature of 25°C yields a predicted growth rate for the pest that is less than half that expected from constant temperatures, because the organism is regularly exposed to suboptimal temperatures as the temperature fluctuates above and below its thermal optimum (Fig. 4B). A similar pattern has been demonstrated experimentally for mosquito vectors (Paaijmans *et al.*, 2013) and several parameters associated with both *Plasmodium* and chytrid fungal transmission (Box 1). This suggests that models for the risk of malaria and chytridiomycosis (the disease caused by the amphibian chytrid fungus) based on mean temperatures might substantially under- or over-estimate transmission at particular temperatures (Paaijmans *et al.*, 2009, 2010; Mordecai *et al.*, 2013; Raffel *et al.*, 2013), with potential consequences for disease-driven host declines (Box 1).

**Box 1. Climate-driven non-linearities, disease, and biodiversity losses**

Non-linear responses of host and/or parasite to temperature can complicate predictions of the effects of climate change on disease risk and thereby threaten conservation efforts. Non-linearities in disease risk arise in at least two ways. First, they arise as a result of integration across generally linear portions of host and parasite temperature performance curves that have opposite slopes (Fig. 3). For example, Paull *et al.* (2012) found a mid-temperature peak in the risk of trematode-induced deformities in the amphibian *Pseudacris regilla*. Fewer deformities at the warmest temperature appear to have been the result of faster host development, and a consequent reduction in the time that hosts spent in early developmental stages where they are vulnerable to deformities. Reduced deformities in the coldest treatment were consistent with an observed decline in parasite infectivity at that temperature. Thus, the combined positive effect of temperature on parasite infectivity and negative effect on host vulnerability may have been responsible for the observed non-linear temperature–disease risk function in this system (Fig. 3). These trematode infections have been associated with mass malformations and reduced survival in amphibians (Johnson *et al.*, 2004), again illustrating the conservation importance of integrating the non-linear physiological responses of both host and parasite to temperature (Rohr *et al.*, 2011a; Mordecai *et al.*, 2013).

Non-linearities can also arise if host, vector, and/or parasite performances across temperature are inherently non-linear, as was shown for the amphibian chytrid fungus, *Batrachochytrium dendrobatidis* (Raffel *et al.*, 2013), which has been implicated in hundreds of amphibian extinctions (Stuart *et al.*, 2004; Wake and Vredenburg, 2008), and the performance of *Plasmodium* spp. and their vector (Paaijmans et al., 2009, 2010, 2013; Mordecai *et al.*, 2013). While the studies on *Plasmodium* focused on human malaria, similar patterns are likely to hold for the ecologically similar avian malaria, which has been implicated in extinctions of several Hawaiian birds (Vanriper *et al.*, 1986; Garamszegi, 2011). Importantly, Mordecai *et al.* (2013) showed that the incorporation of empirically demonstrated non-linearities in parasite development and mosquito performance (e.g. development, reproduction, survival, and bite rate) into a model of malaria transmission yielded an optimal transmission temperature of 25°C, which is considerably lower than the 31°C predicted by previous studies. Given the role of malaria in the declines of bird species (Vanriper *et al.*, 1986; Garamszegi, 2011), this example illustrates how non-linearities might affect predictions for how climate change will affect disease risk for hosts of conservation concern.

Temperature variability might also influence the outcome of host–parasite interactions through differences in the acclimatization rates of hosts and parasites to shifts in temperature (Raffel *et al.*, 2006, 2013; Rohr and Raffel, 2010). Relative to the historical variability to which hosts evolved, global change has modified and is projected to modify climate variability, including changes to monthly variability, diurnal temperature range, and the frequency of El Niño events (Easterling *et al.*, 2000; Raisanen, 2002; Yeh *et al.*, 2009). These changes to climate could theoretically be detrimental to parasites if their hosts or vectors drop to low density during these periods, if their short lifespans make it difficult to persist through unfavourable weather conditions, or if their free-living stages are sensitive to unfavourable conditions. However, the climate variability hypothesis for disease-related declines posits that increased unpredictable climatic variability and extreme events might provide a temporary advantage to pathogens, because they are smaller (fewer cells and processes) and have faster metabolisms than their hosts, thereby allowing them to acclimatize more quickly following a temperature shift (Raffel *et al.*, 2006, 2013; Rohr and Raffel, 2010).

Support for the climate variability hypothesis in driving disease-related declines of amphibians and abalone is growing (Box 2), but several underlying assumptions and the generality of the hypothesis remain to be tested thoroughly (Box 3). Nevertheless, the hypothesis holds great promise for enhancing predictions about how disease risk will be modified by simultaneous changes to both the mean and the variance of climatic conditions and thus how and where conservation efforts related to disease should be targeted. Moreover, most proposed mechanisms for how climate change can generally increase infectious disease are highly controversial, because increases in temperature are expected to cause parasite range shifts rather than expansions (Lafferty, 2009) and possibly just as many scenarios where optimal temperatures of organisms are exceeded as approached (Rohr *et al.*, 2011a). The climate variability hypothesis, however, might offer a plausible mechanism by which global climate change could, on average, cause general increases in infectious diseases, because temperature variability is becoming less predictable, acclimatization of physiological parameters (e.g. cold hardiness) to temperature shifts is a widespread phenomenon observed across ectothermic taxa (Angilletta, 2009), and all parasites are smaller than their hosts and thus should acclimatize more quickly than hosts to temperature shifts. Consequently, climatic variability and predictability might represent under-appreciated links among climate change, disease, and biodiversity losses.

## Metabolic theory of ecology: a tool for linking mean climate and climate variability effects to host–pathogen interactions

We have argued for the need to model simultaneous changes to climatic means and variances in order to predict host–parasite interactions accurately, but we have yet to offer paths forward to do so. We propose that there are at least two general paths forward. The first is to develop species-specific, well-parameterized models that are targeted to particular disease systems of interest. This would facilitate the development of highly resolved predictions of value to public health or conservation. However, such an approach is labour intensive. Alternatively or additionally, efforts can be focused on the development of more strategic models that aim to identify general physiological principles for how climate change will affect host–pathogen interactions. While the benefit of this approach is its generality, it remains unclear how accurate it will be for ‘on-the-ground’ predictions. Pursuit of both approaches in tandem may ultimately represent the most effective path forward.

We submit that the metabolic theory of ecology offers a general modelling framework for predicting how changes to climatic means, variances, and extremes will affect disease risk. The MTE relates an individual organism's metabolic rate (*I*), or its rate of energy uptake and allocation, to its body size (*M*) and the ambient temperature (*T*, in kelvin) using the expression *I* ∝ *M*¾e^−*E*/*kT*^, where *E* is the average activation energy of respiration and *k* is Boltzmann's constant (Brown *et al.*, 2004). For temperature, total metabolism increases exponentially over a ‘normal’ range of temperatures owing to the influence of temperature on chemical reaction rates (although note that, above a critical threshold temperature, enzymes denature and halt reaction rates). There are also extensions of this theory that predict unimodal effects of temperature on physiological processes, such as the Van't Hoff–Arrhenius and Sharpe–Schoolfield models (see modelling section below; Hechinger, 2013; Molnár *et al.*, 2013). For body size, the increase in metabolic rate is allometric (i.e. *M* raised to the ¾ power rather than unity, which would be isometric). This means that the mass-specific metabolic rate (or *I* divided by *M*) decreases to the −¼ power with increases in body size. Stated another way, larger-bodied organisms expend less energy per unit mass relative to smaller-bodied ones, typically owing to increases in heat retention and metabolic efficiency with size. Owing to the importance of metabolic rate in determining both resource uptake and resource allocation, MTE has successfully predicted life history, demographic patterns, and ecosystem processes in taxa ranging from protists to mammals (Brown *et al.*, 2004; Price *et al.*, 2012).

### Metabolic theory of ecology and disease ecology

Although the MTE shows great potential for predicting climate change–disease interactions, thus far, the relatively few studies that have examined components of the MTE in relationship to parasites and body mass have yielded mixed results. In a study of fish parasites, Poulin and George-Nasciemento (2007) reported that parasites within hosts violated the ‘energetic equivalency rule’, which states that the energy use of a population is independent of body size (Damuth, 1987). In a survey of free-living and parasite biomass within estuarine systems, Hechinger *et al.* (2011) reported that including parasites alongside free-living taxa required incorporation of a correction term (which they ascribed to ‘trophic position’). Finally, Hoverman *et al.* (Johnson PTJ, Paull SHP. Unpublished observations) found that parasites within hosts had much lower metabolic rates than expected based on relationships developed for free-living organisms, such that they operated functionally as part of the host rather than as separate organisms. Collectively, these studies suggest that the body size–metabolic rate linkage may be different for parasites than for free-living organisms and emphasize the need for more comparative work incorporating parasitic life histories.

**Box 2. Climatic shifts, acclimatization effects, and declines of amphibians and abalone**

The climate variability hypothesis for disease emergence posits that increased unpredictable climatic variability and extreme events driven by global climate change might provide a temporary advantage to pathogens, because pathogens are almost always smaller than their hosts (fewer cells and processes) and thus have faster metabolisms, possibly allowing them to acclimatize more quickly following a temperature shift (Raffel *et al.*, 2006; 2013; Rohr and Raffel, 2010). Support for the climate variability hypothesis is growing, especially for disease-associated declines of amphibians and abalone. Climate variability was used to explain suboptimal amphibian immunity following particular seasonal shifts in temperature (Raffel *et al.*, 2006). This finding subsequently spurred tests of associations between various climatic predictors, including temperature variability, and the last year in which each of >100 species of harlequin frogs in the genus *Atelopus* were observed. Most of these declines are believed to have been caused by infections with the chytrid fungus, *B. dendrobatidis* (La Marca *et al.*, 2005). After detrending the data to reduce the influence of temporal confounders (Rohr *et al.*, 2008), these analyses revealed that mean climate variables were not nearly as strong predictors of the fluctuations in amphibian declines as were variables representing climatic variability, consistent with recent work revealing that diurnal temperature range, a measure of temperature variability, was predictive of regional and global *B. dendrobatidis* abundance on amphibians (Murray *et al.*, 2011; Rohr *et al.*, 2011b; Liu *et al.*, 2013). Indeed, factors reflecting temperature variability were the only proximate climate variables that were entirely consistent with the spatiotemporal patterns of declines known to be caused by *B. dendrobatidis* (Rohr and Raffel, 2010). The ultimate drivers of these patterns may very well be global El Niño events. A path model showed that El Niño events were associated with greater monthly and daily temperature variability, and this increased variability at these two time scales was associated positively with *Atelopus* declines (Rohr and Raffel, 2010). Importantly, other pathogens associated with amphibian losses, such as the water mold *Saprolegnia ferax*, are also positively associated with the strength of El Niño episodes (Kiesecker *et al.*, 2001).

Recent empirical tests of the climate variability hypothesis support field patterns by showing that frogs that experienced an unpredictable temperature shift, especially an unpredictable temperature drop, had both more *B. dendrobatidis* and greater mortality than frogs that experienced a constant temperature (Raffel *et al.*, 2013). These findings are consistent with a mathematical model showing how temperature variability should affect host–parasite interactions (Raffel *et al.*, 2013). Moreover, drops in temperature were more predictive of *Atelopus* declines than increases in temperature (Raffel *et al.*, 2013), consistent with *B. dendrobatidis* outbreaks generally occurring during cool seasons (Retallick *et al.*, 2004; Kriger and Hero, 2007; Kinney *et al.*, 2011) and with reports that drops in temperature trigger the release of *B. dendrobatidis* zoospores (Woodhams *et al.*, 2008), reduce the ability of amphibians to mount an antimicrobial skin peptide-based immune response, and, instead, induce a more pronounced inflammatory reaction that is associated with higher *B. dendrobatidis* burden (Ribas *et al.*, 2009).

Climatic variation also appears to have driven disease-induced declines of intertidal black abalone (*Haliotis cracherodii*) throughout much of southern California (Ben-Horin *et al.*, 2013). A rickettsial-like disease that causes a condition known as withering syndrome emerged in the 1980s and was associated with mass mortality. Through a series of field and laboratory experiments, it was shown that, similar to the frog–chytrid system, temperature variability increased the susceptibility of black abalone to infection (Ben-Horin *et al.*, 2013). Disease expression then occurred once water temperatures exceeded thresholds modulating withering syndrome (Ben-Horin *et al.*, 2013). These studies on temporal variability in temperature and infections of frogs and abalone emphasize the importance of acclimatization effects for understanding climate-driven declines associated with disease and highlight the importance of physiology for elucidating biodiversity losses and targeting conservation efforts (Li *et al.*, 2013).

One of the reasons why the body size–metabolic rate relationship might appear to be different for parasites compared with free-living organisms is because it is not entirely clear what mass (*M*) term to use for parasites. There are reasonable arguments for using the following parameters: (i) the mass of individual parasites; (ii) the mass of the entire parasite population within a host; (iii) the mass of the host, which could control parasite metabolism; or (iv) some relationship between host and parasite biomass that might depend on whether the host is an endo- or ectotherm. Cable *et al.* (2007) found that, across a range of pathogen and host types, the time to pathology scaled with the mass of the host multiplied by a scaling term, or *cM*^1/4^. How *c* related to pathogen mass was not explored, however, and in general, more empirical data and syntheses examining the influence of temperature, body size (host and pathogen), and pathogen transmission mode are sorely needed to evaluate these issues thoroughly (Box 3). Through a series of models validated with data, Hechinger (2013) emphasized the importance of energy constraints (rather than space constraints) in affecting parasite-carrying capacity and maximal energy flux within hosts. According to Hechinger (2013), incorporation of both host and per capita parasite mass into an MTE theoretical framework allows a more direct prediction of total parasite biomass within hosts. Owing to an overall shortage of empirical data, however, there remain few opportunities to test these principles and evaluate whether the metabolic relationships for parasites differ from those for non-parasitic species, which is an important prerequisite before climate change predictions could be used to promote conservation.

**Box 3. Outstanding questions**

- How often do non-linearities occur in the differential temperature-dependent performance curves of interacting species and what are the shapes of these curves? That is, how often and by how much are our predictions off if we assume linear responses?
- How important is climatic variability relative to mean temperature and precipitation in dictating the outcome of host–parasite interactions and conservation physiology?
- Is the underlying assumption of the temperature variability hypothesis of parasitism, that parasites acclimatize to climatic shifts more quickly than their hosts, generally true? If so, do acclimatization responses scale with size based on the ¾ power law, as do other metabolic processes? Can we predict the advantage that a parasite has with a given climatic shift simply by knowing the difference in size between host and parasite?
- Is the acclimatization response general across ectothermic hosts and pathogen species? Are there any traits of hosts and parasites other than size that might explain any variability in acclimatization (see below)?
- What is the functional relationship between the magnitude and frequency of climatic shifts and the outcome of host–parasite interactions?
- Does the direction of a temperature shift influence host–parasite interactions and, if so, is it predictable by determining the thermal performance curves of the two species?
- Is all variability the same or does the outcome of host–parasite interactions depend on the predictability of the variation?
- Can the traits of hosts or parasites predict how important variability will be to their interactions? For instance:
  - Endo- vs. ectothermic hosts
  - Endo- vs. ectoparasites
  - Parasites with free-living stages or not
  - Tropical (climatically stable) vs. temperate (climatically variable)
- Are species from tropical regions more susceptible to effects of thermal variability, because climates are more stable than for species from temperate regions?
- To what extent can species living in heterogeneous environments use behavioural thermoregulation to mitigate effects of unfavourable climate shifts?
- What are the context dependencies of the acclimatization effect? For instance, are there interactions between temperature variability and precipitation, or between climate and other factors (e.g. location/latitude)?
- What are the mechanisms of the acclimatization effect? Does acclimatization generally affect resistance or tolerance to parasites, or both?
- Different components of physiology acclimatize at different rates (e.g. immunity vs. metabolic rate); how does this affect predictions for how variability in climatic conditions affects host–parasite interactions?
- Can we link actual global climate change to acclimatization effects and, if so, does it offer a general climate-based hypothesis for disease emergence?

One of the most exciting potentials for applying the MTE to host–pathogen interactions involves more deeply exploring the temperature-dependency component of vital rates, which thus far has received surprisingly little attention in disease research. A groundbreaking new study by Molnár *et al.* (2013) linked MTE host–macroparasite models to understand the ‘fundamental thermal niche’ of parasites and how their fitness (e.g. *R*_0_, or the basic reproductive number of parasites) varies with changes in temperature. Using arctic nematodes and their mammalian hosts as their empirical foundation, the authors developed temperature-dependent values for parasite mortality and development rate during their free-living periods (which is when they are infectious to roaming mammals). They then developed a temperature-dependent value of *R*_0_, a focal parameter of epidemiologists and studies of disease dynamics, that accounted for trade-offs between development and mortality expected to occur with changing temperatures (i.e. to identify the net effects). This model offered both general predictions about how infection patterns will shift in different warming scenarios and, when parameterized to the nematode system, specific predictions that corresponded well with available empirical data. It also drew attention to the central importance of identifying the threshold temperatures at which parasite development ceases or mortality occurs; values that are either not measured in many empirical studies or are often presented in an unstandardized way.

### Using the metabolic theory of ecology to model host–parasite interactions in a changing temperature environment

To illustrate how a host–parasite interaction could be described using thermal biology theory, we focus on the simple case of a directly transmitted microparasite infecting an ectothermic host and build upon the MTE-based models of DeLeo and Dobson (1998), Morand and Poulin (2002), Molnár *et al.* (2013), and Hechinger (2013). Once a host is exposed to the parasite, we assume that the parasite's replication rate within the host, λ_*T*_, is a function of the temperature-dependent infectivity of the parasite, *i*_*T*_, and resistance of the host to infection, *r*_*T*_, such that:
(1)
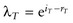



Infectivity here represents the within-host parasite population growth rate in ideal conditions (i.e. in the absence of a host immune response), and resistance is the ability of the host to reduce this population growth rate. The parasite population grows (λ > 1) if *i*_*T*_ > *r*_*T*_, and declines (λ < 1) if *r*_*T*_ > *i*_*T*_. To derive equations for infectivity and resistance as a function of temperature, we used the Sharpe–Schoolfield model for temperature-dependent performance of a physiological parameter, which assumes that performance scales with the Boltzmann factor, 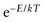 and that reversible deactivation of rate-controlling enzymes occurs at high and low temperatures, *T*^*H*^ and *T*^*L*^ (Molnár *et al.*, 2013). This approach gives us:
(2a)
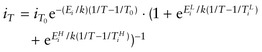

(2b)
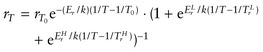

where 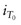 and 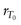 are infectivity and resistance measured at the standardization temperature *T*_0_, *E*_*i*_ and *E*_*r*_ are the activation energies for infectivity and resistance, 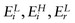, and 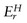 are the low- and high-temperature deactivation energies for infectivity and resistance, 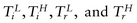 are the low- and high-temperature thresholds for infectivity and resistance (in kelvin), and *k* = 8.62 × 10^−5^ is Boltzmann's constant. Metabolic theory and empirical measurements indicate that activation energies are similar across different physiological processes and organism taxa, allowing us to simplify the model (*E*_*i*_ ≈ *E*_*r*_ ≈ 0.65; Molnár *et al.*, 2013). Metabolic theory is less informative regarding deactivation energies, but we assumed that 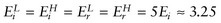, after Molnár *et al.* (2013). For parasites that can be cultured, the replication rate of the parasite in culture at the standardization temperature *T*_0_ (any temperature within the normal range for infection) could be used as a proxy for 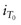, with resistance 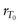 estimated as the decrease in parasite replication at *T*_0_ while growing in or on the host, relative to growth in culture. Note that temperature is modelled in kelvin, but is described hereafter in degrees celsius for clarity.

As an example of how this model could be implemented for a real parasite–host system, we estimated parameters for temperature dependence of the pathogenic fungus *B. dendrobatidis* growing on a susceptible Cuban treefrog (*Osteopilus septentrionalis*). We calculated low- and high-temperature thresholds (*T*^*L*^ and *T*^*H*^) based on estimates of the critical thermal maximum and minimum for *B. dendrobatidis* growth (CT_min_ = 0°C; CT_max_ = 30°C) and Cuban treefrog survival (CT_min_ = 6.4°C; CT_max_ = 39.0°C; John-Alder *et al.*, 1988; Rohr *et al.*, 2008). The range of temperatures over which an organism can perform a particular physiological function (e.g. development) is generally narrower than the range over which an organism can survive (Molnár *et al.*, 2013), so we assumed that *T*^*L*^ and *T*^*H*^ for infectivity and resistance were 5°C higher and lower than CT_min_ and CT_max_, respectively 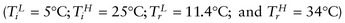. We estimated 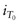
 = 0.78, based on the average growth rate of *B. dendrobatidis* in culture at *T*_0_ = 20°C, as measured by Piotrowski *et al.* (2004) and Woodhams *et al.* (2008) and analysed for population growth rates by Rohr *et al.* (2008). Model predictions were generated using the program R (R Development Core Team, 2010). Predicted values for *i*_*T*_ and λ_*T*_ were consistent with published patterns of temperature dependence for this parasite in culture and on Cuban treefrogs (Fig. 2A and B; Rohr *et al.*, 2008; Raffel *et al.*, 2013).

To show how this model could be incorporated into a dynamic model of a simple microparasite system, we started with a standard *SI* (susceptible–infected) model assuming density-independent growth of the host population, and that infected hosts become susceptible again following recovery. In this model, 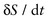 and 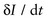 represent the rates of change in the sizes of the susceptible and infected host populations, respectively. We then added our temperature-dependent parameter λ_*T*_ to this model, assuming that parasite transmission and virulence are both directly proportional to λ_*T*_ and that host recovery is inversely proportional to λ_*T*_, such that:
(3a)
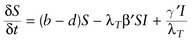

(3b)
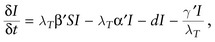

where *b* and *d* are the background birth and death rates, and β′, α′, and γ′ are constants controlling the effects of λ_*T*_ on transmission, virulence, and recovery, respectively. From this model we can set 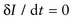 to derive the basic reproductive ratio *R*_0_, a critical parameter indicating the expected number of new infections resulting from one infected individual in a population of susceptible individuals, as a function of temperature, as follows:
(4)
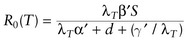


To illustrate the relationship between λ_*T*_ and *R*_0_(*T*), we selected arbitrary parameter values of β′ = 0.001, α′ = 0.2, γ′ = 0.2, *d* = 0.1, and *S* = 500 (Fig. 2B). This set of parameters predicts that *R*_0_(*T*) will be >1 for temperatures below 25°C (Fig. 2B), consistent with laboratory and field data on frog infections and population declines (Rohr and Raffel, 2010; Raffel *et al.*, 2013). Note that this model formulation constrains *R*_0_(*T*) to be positively related to λ_*T*_ over the entire range of possible parameter values.

We can incorporate thermal acclimatization responses into this model by allowing the low- and high-temperature limits for infectivity and resistance 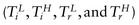 to vary according to the temperature to which the host or parasite is acclimatized (*T*_accl_). If we assume that these parameters vary linearly with acclimatization temperature, the value of each parameter for a given acclimatization temperature can be estimated by measuring each parameter at two acclimatization temperatures, *T*_1_ and *T*_2_. For example, 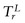 as a function of the host's acclimatization temperature would be determined using the following equation:
(5)
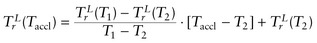



To model changes in host and parasite acclimatization status through time (*t*) in a changing temperature environment, we assumed that each organism is acclimatized to the weighted average of temperature (*T*) over the past ψ time units, with greater weight given to more-recent temperatures, as follows:
(6a)
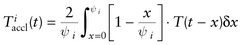

(6b)
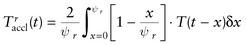

where ψ_*i*_ and ψ_*r*_ represent the time it takes for the parasite and host to acclimatize, respectively. Here too, metabolic theory provides guidance for parameter estimation, because the time it takes for organisms to acclimatize following a temperature shift (ψ) is likely to scale with organism mass, with larger organisms taking longer to acclimatize than smaller organisms (Raffel *et al.*, 2013). If we assume that ψ scales with mass in a similar manner to organism development times, then
(7)
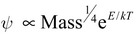


Assuming that this relationship holds for parasitic organisms, this formula predicts that parasites should acclimatize faster than hosts, with a host-to-parasite acclimatization-time ratio proportional to the quarter power of the host-to-parasite mass ratio. Whether acclimatization rates actually scale with body size, however, remains to be tested (see above and Box 3). If they do, the size difference between ectothermic hosts and parasites might predict how long parasites have an advantage, given their theoretically faster acclimatization rates.

**Figure 5:**
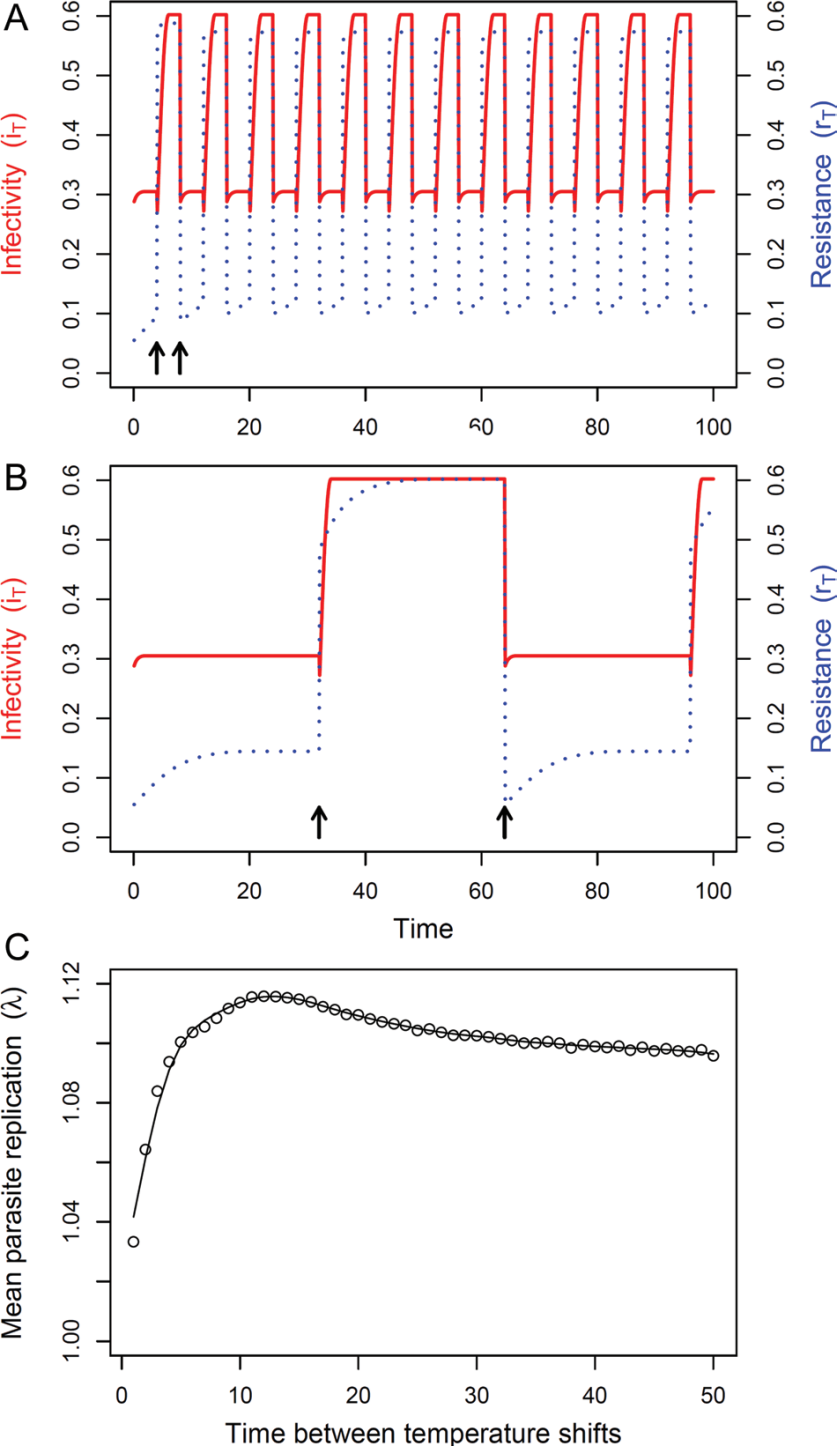
Model predictions for changes in parasite infectivity (continuous red line) and host resistance (dotted blue line) in a changing temperature environment, with temperature shifts between 25 and 10°C occurring every 4 days (A) or every 32 days (B). Arrows indicate the first two time points when temperature shifts occurred (25 → 10 and 10 → 25°C). (C) Mean parasite replication rates (λ) over 18 000 time steps, for different temperature-shift frequencies ranging from daily shifts to shifts occurring every 50 days. The curve is a smoothing spline (function ‘smooth.spline’) with a smoothing parameter of 0.5.

As an example of how this model could be applied to predict parasite growth rates in or on hosts in a variable temperature environment, we applied the model parameters used to generate Fig. 2 (see above) to a model of parasite replication rates in a changing temperature environment. We generated time series of shifting temperatures (shifts between 10 and 25°C), ranging from shifts occurring every day to shifts occurring every 50 days. These time series were then converted into continuous functions using linear interpolation (function ‘connector’ from the ‘mosaic’ package; Pruim *et al.*, 2012), which were then used to generate model predictions for infectivity (*i*_*T*_), resistance (*r*_*T*_), and parasite replication rate (λ_*T*_) at each time point. The high and low deactivation temperatures used to generate Fig. 2 (see above) were assumed to be for warm-acclimatized parasites and hosts (*T*_accl_ = 25°C), whereas deactivation temperatures were 4 and 8°C lower for cold-acclimatized parasites and hosts, respectively (*T*_accl_ = 5°C). Hosts and parasites were assumed to take 20 and 2 days, respectively, to acclimatize fully to a new temperature (ψ_*r*_ = 20; ψ_*i*_ = 2), and were acclimatized to 25°C at the start of each time series. This temperature-explicit model predicted that infectivity and resistance would both increase with time following a temperature shift (Fig. 5A and B) and that parasite replication rates would be maximal with intermediate temperature shift frequencies (Fig. 5C), as postulated by Raffel *et al.* (2013).

Importantly, this modelling framework that builds upon the models of Molnár *et al.* (2013) should facilitate the prediction of the magnitude and direction of a change to disease risk in response to known or predicted changes in both climatic means and variances. This should then improve predictions of climate change–disease interactions, as well as the effectiveness of conservation measures. However, even in the absence of strong acclimatization effects, the incorporation of temperature variability into disease models remains important, because models that simply extrapolate from mean climate values can provide erroneous predictions compared with those that integrate over realistic temperature ranges (see Mordecai *et al.*, 2013).

## Conclusions

Here we argued for greater integration of physiology and disease ecology to provide better understanding of climate- and disease-associated host declines. We recommend the incorporation into predictive mathematical models both non-linear responses to climate and differences in the acclimatization responses of hosts and parasites to climatic shifts, because both have recently been shown to have important consequences for disease dynamics associated with host declines. In particular, rather than simply modelling climatic means, predictive models should move towards capturing changes in climatic variances and extremes, given that these changes are a hallmark of climate change (Easterling *et al.*, 2000; Meehl *et al.*, 2000, 2009; Raisanen, 2002; Kunkel *et al.*, 2003). The metabolic theory of ecology offers intriguing promise for predicting host–parasite interactions within an environmental context. Given that parasites are always substantially smaller than their hosts and that body size is a reliable proxy of metabolic rate, the MTE should help to integrate physiological mechanisms and large-scale spatiotemporal processes to enable successful prediction of how changes in climatic means, variances, and extremes will affect host–parasite interactions. We hope that our mathematical model, based on the integration of metabolic theory and physiological mechanisms, provides the scaffolding to enable more successful prediction of how host–parasite interactions will respond to changes in climatic means, variances, and extremes. However, the success of these predictive models will depend on addressing many of the outstanding questions regarding the relationships between climate, physiology, and host–parasite interactions (Box 3). Addressing these pressing knowledge gaps and using this new information to improve climate–disease models should improve the capacity to predict how climate change will affect disease risk for species of conservation concern.
